# Effect of a direct-fed microbial (10-G Armor) on feedlot performance, carcass characteristics, and prevalence of *Salmonella* in fed-beef heifers

**DOI:** 10.1093/tas/txac073

**Published:** 2022-05-26

**Authors:** Lauren M Mayer, Kevin Martens, Alyssa B Word, Ben P Holland, Loni L Lucherk, Ty E Lawrence, Travis C Tennant

**Affiliations:** Beef Carcass Research Center, West Texas A&M University, Canyon, TX 79016, USA; Life Products, Inc., Norfolk, NE 68701, USA; Cactus Research, Amarillo, TX 79101, USA; Cactus Research, Amarillo, TX 79101, USA; Beef Carcass Research Center, West Texas A&M University, Canyon, TX 79016, USA; Beef Carcass Research Center, West Texas A&M University, Canyon, TX 79016, USA; Beef Carcass Research Center, West Texas A&M University, Canyon, TX 79016, USA

**Keywords:** carcass, direct-fed microbial, heifers, performance, prevalence, *Salmonella*

## Abstract

Crossbred beef heifers (*N *= 1,394; initial shrunk body weight [BW] 291 ± 9.9 kg) were used to investigate the efficacy of 10-G Armor (Life Products, Inc., Norfolk, NE; 10-G) upon feedlot performance, carcass characteristics, and fecal and subiliac lymph nodes *Salmonella* prevalence. Heifers were blocked by day of arrival and allocated to 1 of 20 pens (*N* = 70 heifers/pen) and assigned one of two treatments (10 pens/treatment): no direct-fed microbial (CON) or 2 g/heifer/d of *Lactobacillus acidophilus, Enterococcus faecium, Pediococcus pentosaceus, Lactobacillus brevis* and *Lactobacillus plantarum*, respectively (Life Products, Inc., Norfolk, NE; 10-G). Twenty-four animals were randomly selected from each pen for *Salmonella* sampling. Recto-anal mucosal swab samples (RAMS) were obtained at initial processing and harvest; subiliac lymph nodes were collected at harvest. In addition, pen surface fecal pats were collected and composited by pen (10 pats per composite, 5 composites per pen) on days 0, 52, 120, and 192. Data were analyzed as a generalized complete block design, and pen served as the experimental unit. No differences were observed in live growth performance metrics (*P* ≥ 0.55). Yield grade distributions did not differ between treatments (*P* ≥ 0.62); however, cattle fed 10-G tended (*P* = 0.06; 14.6% vs. 18.9%) to have fewer USDA Select carcasses and more (*P* = 0.09; 73.6% vs. 78.0%) USDA Choice carcasses. Cattle fed 10-G tended (*P* = 0.10; 9.2% vs. 12.3%) to have fewer liver abscesses and had fewer (*P* = 0.04; 5.3% vs. 8.5%) severe liver abscesses. *Salmonella* prevalence of RAMS did not differ between treatments at initial processing (*P* = 0.97; CON = 11.6%, 10-G = 11.5%) or at harvest (*P* = 0.91; CON = 99.0%, 10-G = 98.6%); however, RAMS differed (*P* < 0.01) in *Salmonella* prevalence between the two collection times. Cattle fed 10-G had a lower frequency of *Salmonella* positive lymph nodes (*P* = 0.01; CON = 15.8%, 10-G = 7.4%) than CON. However, *Salmonella* log (mpn/g) of lymph nodes did not differ between treatments at harvest (*P* = 0.34; CON = 0.73, 10-G = 0.34). These data indicate that cattle fed 10-G have decreased rates of severe liver abscesses without altering live animal performance or carcass characteristics. Supplementation of 10-G significantly reduced the prevalence rate of *Salmonella* recovered from the subiliac lymph nodes. The factors responsible for the observed difference in the effects of 10-G on *Salmonella* warrant further investigation

## INTRODUCTION

An estimated 48 million cases of foodborne illness occur annually in the United States; non-typhoidal *Salmonella* is the leading cause of bacterial foodborne illnesses with 1.35 million cases, 26,500 hospitalizations, and 420 deaths (CDC, 2019). *Salmonella* is a naturally occurring bacterial pathogen historically associated with poultry (Whyte et al., 2002; Parveen et al., 2007; Foley et al., 2008) eggs (Jones et al., 1995, 2012; Singh et al., 2010) and produce (Wells and Butterfield, 1997; Quiroz-Santiago et al., 2009; SantʹAna et al., 2011). Studies have shown that beef products are also susceptible to *Salmonella* contamination (Rose et al., 2002; Zaidi et al., 2008; Sallam et al., 2014). Lymph nodes in beef cattle are known to harbor *Salmonella* (Samuel et al., 1980; Arthur et al., 2008; Brown et al., 2020); furthermore, the ability to remove all lymph nodes from a beef carcass is impractical. As a result, lymph nodes may be incorporated into ground beef trimmings, thus increasing the risk of *Salmonella-*contaminated ground beef (Arthur et al., 2008; Bosilevac et al., 2009; Koohmaraie et al., 2012). *Salmonella* prevalence differs seasonally; frequency peaks during the summer through early fall and troughs during the winter (Barkocy-Gallagher et al, 2003; Dargatz et al., 2003; McEvoy et al., 2003). Feedyard location also affects *Salmonella* prevalence; southern regions have a higher prevalence of *Salmonella* than northern regions (Dargatz et al., 2003; Rivera-Betancourt et al., 2004; Haneklaus et al., 2012). Additionally, cattle type affects *Salmonella* prevalence; feedlot cattle are more frequent carriers than cull cows and bulls (Gragg et al., 2013a; Webb et al., 2017). Fed Holstein steers had a higher prevalence of *Salmonella* than beef-type steers, whereas cull dairy cows had a higher prevalence than range cows (Herrick, 2022).

Effective intervention technologies implemented to decrease *Salmonella* include vaccines (Edrington et al., 2013; Cernicchiaro et al., 2016) and various feed additives, including seaweed extract (Braden et al., 2004), tylosin (Amachawadi et al., 2017), and direct-fed microbials (DFMs; Stephens et al., 2007; Vipham et al., 2015; Brown et al., 2020). Direct-fed microbials are commonly used in the industry to improve performance and may reduce the pathogenic load in cattle. Shedding of *Salmonella* has been reported to be reduced by DFMs (Stephens et al., 2007; Vipham et al., 2015; Brown et al., 2020), while others reported no difference (Tabe et al., 2008). Bacterial DFMs have been reported to reduce pathogenic microorganisms in cattle via modifying the balance of intestinal microorganisms, competitive attachment to the intestinal mucosa, influencing gut permeability, formation of antimicrobial proteins or bacteriocins, and modulating immune function (Krehbiel et al., 2003). Yet, the effects of a mixture of *Lactobacillus acidophilus, E. faecium, P. pentosaceus, L. brevis*, and *L. plantarum* on growth performance, carcass merit, and *Salmonella* prevalence of fed-beef heifers have not been reported, even though there is evidence that these microbes may have beneficial properties in this regard when fed individually (Luebbe et al., 2013). The objectives of this study were to evaluate the inclusion of *L. acidophilus, E. faecium, P. pentosaceus, L. brevis*, and *L. plantarum* in fed-beef heifers on growth performance, carcass characteristics, and *Salmonella* prevalence.

## MATERIALS AND METHODS

The feeding portion of this experiment was conducted at a commercial feedyard in the Texas panhandle. All experimental procedures were approved by the Institutional Animal Care and Use Committee at West Texas A&M University (#2020.02.003).

### Cattle Processing and Experimental Design

Crossbred beef heifers (*N* = 1,925) were received at a commercial feedyard in the Texas Panhandle between February 28, 2020, and March 14, 2020, from Texas, Alabama, and Tennessee. Prior to initial processing, cattle were penned together by source and were provided ad libitum access to water and prairie grass hay. During initial processing, heifers were excluded from the trial if their initial body weight (BW) deviated more than 68 kg from the average pay weight and were deemed unfit due to illness, lameness, or pregnancy. Heifers were initially implanted with Revalor-IH (Merck Animal Health, Summit, NJ) and at re-implant (77-79 DOF) received Revalor-200 (Merck Animal Health). Heifers were administered Titanium 3 (Elanco Animal Health, Indianapolis, IN) and Nasalgen IP (Merck Animal Health) for viral respiratory pathogens. Internal and external parasites were controlled through the administration of Synanthic (Boehringer Ingelheim, Duluth, GA) and Dectomax Injectable (Zoetis, Parsippany, NJ). Heifers were identified with visual ear tags that contained the last three digits of the lot associated with the pen as well as an individual number specific to the animal.

In total, 1,400 heifers with an initial BW of 291 ± 9.9 kg were enrolled in this study in a generalized complete block design with time of arrival as blocking factor and pen as the experimental unit. Each arrival block (*N = *5) contained four pens with two replications of each dietary treatment. Pens (*N = *20) each housed 70 heifers. Animals were randomly assigned to pens within blocks using a computer-generated schedule. Heifers were randomly allocated to one of two treatments: 0 g/animal/d (CON) or 2 g/animal/d (10-G) of 10-G Armor (Life Products, Inc., Norfolk, NE) to provide 1 billion colony forming units per animal per day of *L. acidophilus, E. faecium, P. pentosaceus, L. brevis*, and *L. plantarum. *Within each pen, 24 candidate animals were randomly identified for longitudinal *Salmonella* sampling. Individual BW was collected at initial processing and re-implant; pen BW was collected on day 0 and prior to harvest using a platform scale (Model 7531, Mettler-Toledo, Columbus, OH) prior to the morning feeding.

A 2% pencil shrink was applied to the initial BW, whereas a 4% pencil shrink was applied to the final BW due to differences in proportional BW. After randomization but prior to day 0, five heifers assigned to the CON treatment died. The cause of death was liver failure (1), peritonitis (2), and bovine respiratory disease (2). Also prior to day 0, one heifer was removed from a 10-G pen due to being pregnant. Upon arrival, all cattle received a starter diet (Table 1) that consisted of RAMP (Cargill Corn Milling, Bovina, TX), and hay was top-dressed for the first 3 d. Cattle were then transitioned to a finishing diet in which RAMP was reduced every 2 to 4 d at a 10% to 15% rate. Both starter and finishing diets included monensin (Rumensin, Elanco Animal Health) and tylosin (Tylan, Elanco Animal Health). Finishing diets also included melengestrol acetate (HeifermaX 500, Elanco Animal Health). In addition, ractopamine hydrochloride (Optaflexx, Elanco Animal Health) was fed for the final 35 d prior to slaughter. Inclusion of micro-nutrients occurred via a Micro Machine (Micro Technologies, Amarillo, TX) and were added directly to each feed batch. 10-G Armor was dispensed independently from a Micro Machine (Micro Technologies) into the ration after it was loaded into the delivery truck (Roto-Mix, Dodge City, KS) and mixed for 3 min.

**Table 1.** Ingredient formulation and analyzed composition of starter and finishing diets^*a*,*b*,*c*^

	Dietary treatment			
	STARTER		FINISHER	
Item	CON	10-G	CON	10-G
Ingredient, %				
RAMP^*d*^	100.00	100.00	—	—
Steam-flaked corn	—	—	58.98	58.96
Wet distillers grain with solubles	—	—	13.91	13.90
Sweet bran plus^*e*^	—	—	18.29	18.31
Yellow grease fat	—	—	1.44	1.45
Cotton burrs or corn stalks	—	—	7.34	7.33
Starter supplement^*f*^	0.03	0.03	—	—
Finisher supplement^*g*^			0.03	0.05
Nutrient composition, %				
Diet DM, %	65.30	64.20	64.20	63.70
Crude protein, %	21.60	21.40	15.50	15.40
Nonprotein nitrogen compounds, %	0.90	1.00	1.30	1.30
Neutral detergent fiber, %	39.70	39.60	23.70	22.10
Crude fiber, %	3.70	3.60	5.10	5.10
Ca, %	1.30	1.40	0.75	0.78
P, %	0.90	0.90	0.49	0.49
Mg, %	0.40	0.40	0.23	0.23
K, %	1.50	1.60	0.83	0.83

^*a*^Treatments included no DFM contained in the diet (CON) and a diet containing *L. acidophilus*, *E. faecium*, *P. pentosaceus*, *L. brevis*, and *L. plantarum* fed at 2 g/heifer/d providing 1 × 109 CFU (10-G) (Life Products, Inc., Norfolk, NE).

^*b*^All values except DM on a DM basis.

^*c*^Analysis and calculation performed by Servi-Tech Laboratories, Amarillo, TX.

^*d*^Complete starter feed (Cargill Inc., Blair, NE).

^*e*^Wet corn gluten feed (Cargill Inc., Blair, NE).

^*f*^Grower supplement formulated to supply 22.04 mg/kg Rumensin (Elanco, Indianapolis, IN) and 11.02 mg/kg Tylan (Elanco, Indianapolis, IN).

^*g*^Finisher supplement formulated to supply 46.30 mg/kg Rumensin (Elanco, Indianapolis, IN) and 11.02 mg/ kg Tylan (Elanco, Indianapolis, IN), 544.31 IU/kg Vit A, 54.4 IU/kg Vit D, and 44.09 melengestrol acetate.

### Sample and Data Collection

A longitudinal design was used to investigate *Salmonella* shedding, with 24 candidate animals randomly selected from each pen. Recto-anal mucosal swab samples (RAMS) were collected during initial processing (day −2) and at harvest. A sterile foam-tipped applicator swab (FecalSwab, COPANUSA, Murrieta, CA) was inserted 3 to 5 cm into the recto-anal canal junction of each designated heifer. The swab was then placed into a sterile sample bag (WhirlPak, Nasco, Modesto, CA) that was labeled with a sample number that was correlated back to the heifer ID and sealed.

Composite fecal pat samples were collected from each pen on days 0, 52, 120, and 192. Each composite sample represented 10 individual fecal pats; five composite samples were collected per pen. Sample bags were labeled with the appropriate pen number and sample day.

Heifers were fed an average of 192 d (range of 183 to 204 d) prior to being transported 92 km to a commercial beef processor (USDA Establishment #245E) for harvest. Carcass data were collected by trained personnel from the West Texas A&M University—Beef Carcass Research Center (Canyon, TX). Ear tags were individually recorded and assigned an individual identification by West Texas A&M University personnel. Livers were scored using a modified Elanco Liver Check System (Brown and Lawrence, 2010) in which abscesses were evaluated based on severity (edible = no abscesses, A−  = 1 or 2 small abscesses, A = 2 to 4 small active abscesses, A+ = 1 or more large active abscesses, A + Adhesion = liver adhered to the gastrointestinal tract, and A + Open = open liver abscesses). Additionally, other liver abnormalities including telangiectasis, cirrhosis, flukes, and contamination were recorded. Individual lungs were evaluated to determine the presence and severity of lung lesions, interlobular adhesions, and plural adhesions, and missing lobes were recorded. Lung scores were N = normal; 1 = presence of mycoplasma-like lesion > 15%; 2 = plural adhesions, a portion of the lung missing, or a combination of these affecting <25% of lung tissue; 3 = plural adhesions, a portion of lung missing, or a combination of these affecting >25% to <50% of lung tissue; 4 = plural adhesions, a portion of lung missing, or a combination of these affecting >50% to <75% of lung tissue; and 5 = plural adhesions, a portion of lung missing, or a combination of these affecting >75% of lung tissue. Lungs that were contaminated, inflated, or skipped received a C, I, or S score, respectively. Hot carcass weight (HCW) was recorded on the harvest floor. The left or right subiliac lymph node (*n *= 429) was collected from each sampled animal at harvest. Lymph nodes were excised, kept intact, and encased in fat, placed in a bag with a label corresponding to the sample animal. All samples were placed on wet ice and shipped to Food Safety Net Services (San Antonio, TX) for diagnostic analysis. Carcass characteristics (marbling, quality grade, 12th-rib subcutaneous fat depth, longissimus muscle area [LMA], and yield grade) were obtained from USDA camera data.

### *Salmonella* Analysis

Upon arrival at Food Safety Net Services, 10 g of a composite fecal sample was weighed and inserted into a sterile Whirl Pak bag with 90 mL of buffered peptone water. Samples were hand massaged for 30 s to create a homogenous sample. Sample liquid of RAMS was directly transferred to the first well of plates.

Lymph node samples were trimmed of excess fat and fascia and submerged into boiling water for 3 to 5 s to rid the lymph node of any *Salmonella* on the exterior surface. Lymph nodes were then individually placed into stomacher sample bags and weighed, manually pulverized using a rubber mallet, and enriched with 80 mL of tryptic soy broth through incubation at 25 ^°^C for 2 h and then 42 ^°^C for 12 h (Brichta-Harhay et al., 2008; Gragg et al., 2013b). Each sample was replicated three times and serially diluted eight times onto a 96-well plate with four samples per well pin. One milliliter of aliquot was put into each well pin with a serial dilution of 10^−8^ through 10^−1^. The well pin plate was then covered and incubated at 37 °C for 24 h. After incubation, a 96-well pin replicator was used to transfer growth from incubated plates to 1 mL Rappaport-Vassiliadis broth aliquots. The replicated well pins were covered and incubated at 42 °C for 24 to 48 h. After incubation, all samples that were indicative of growth changed colors and were streaked onto xylose lysine deoxycholate agar plates and incubated at 35 °C for 24 h. After 24 h, plates that had colony growth were presumed positive for *Salmonella* and underwent serological confirmation with Poly O antisera to confirm *Salmonella*.

### Statistical Analysis

The GLIMMIX procedure of SAS (version 9.4, SAS Inst. INC., Cary, NC) was used to model the fixed effect of dietary treatment utilizing block as a random effect and pen as the experimental unit. Means were generated using the LSMEANS option and separated using the PDIFF option. Repeated measures were used to analyze *Salmonella* prevalence and concentration across days on feed using the unstructured covariance structure. Nominal data were analyzed as a series of binomial distributions; treatment proportions and standard errors were calculated using the ILINK option. Differences were considered significant at α ≤ 0.05, and trends were noted at 0.05 < α ≤ 0.10.

## RESULTS AND DISCUSSION

Growth performance data are presented in Table 2. Supplementation with 10-G did not affect growth performance as average daily gain (ADG), dry matter intake (DMI), gain:feed (G:F), and final BW did not differ (*P* ≥ 0.63) between treatments. Likewise, no differences (*P* ≥ 0.55) were observed for morbidity or mortalities and removals. Other studies in which 10-G was supplemented to cattle reported similar results for ADG, DMI, F:G, and final BW (Neuhold et al., 2012; Luebbe et al., 2013; Kenney et al., 2015). Live performance results of cattle supplemented with other bacterial DFM are variable. Numerous studies have reported that bacterial DFM supplementation did not alter ADG, G:F, or DMI. Kenney et al. (2015) reported no differences in DMI, ADG, and growth efficiency between DFM and CON; however, performance differences between differing DFM strains were observed for final BW, ADG, and G:F. Cull et al. (2015) reported no difference in DMI between control cattle and those supplemented with a DFM. Similarly, Kenney et al. (2015) found no improvements in growth performance for DFM supplementations; however, they reported differing HCW and ADG among differing DFM strains. Huck et al. (2000) studied the effects of phase feeding of bacterial DFM on the growth performance of finishing heifers and reported no difference in daily gain, DMI, or feed efficiency. Other research by McPeake et al. (2002) reported that between non-supplemented steers and steers receiving diets inoculated with DFM, steers fed a DFM had a greater final live weight, overall ADG, and overall DMI. 

**Table 2.** Live growth performance of heifers fed 10-G

	TRT^*a*^			
Item	CON	10-G	SEM	*P-*value
*n* pens	10	10	—	—
Initial BW, kg	290.9	291.0	9.9	0.95
Final BW, kg	543.0	545.3	8.9	0.79
ADG, kg	1.37	1.38	0.02	0.69
DMI, kg	8.89	8.98	0.27	0.63
G:F	0.154	0.155	0.005	0.81
Morbidity, %	9.57	9.37	—	0.90
Mortalities and removals, %	6.17	6.98	—	0.55

^*a*^Treatments included no DFM contained in the diet (CON) and a diet containing *L. acidophilus*, *E. faecium*, *P. pentosaceus, L. brevis*, and *L. plantarum* fed at 2 g/heifer/d providing 1 × 109 CFU (10-G) (Life Products, Inc., Norfolk, NE).

### Liver and Lung Outcomes

The percentage of edible livers (Table 3) for 10-G and CON cattle were 84.54% and 81.53%, respectively, and did not differ (*P = *0.17) between treatments. Conversely, heifers fed 10-G tended (*P = *0.10) to have a lower frequency of abscesses (9.23%) when compared with CON (12.26%). The rates of edible or abscessed livers observed in the current study are similar to those reported by Brown and Lawrence (2010) and Herrick (2022). The total severe abscess (A+, A + Open, A + Adhesion, A + Adhesion/Open) incidence rate differed between dietary treatments (*P = *0.04) with CON cattle having 8.51% severely abscessed livers, whereas 10-G cattle had 5.27% severely abscessed livers. Severely abscessed incidence rate for CON was numerically higher than the 6.0% incidence rate reported by the National Beef Quality Audit (Eastwood et al., 2017). Livers condemned for reasons other than abscesses (flukes, telangiectasis, contamination) did not differ (*P ≥ *0.28).

**Table 3.** Liver and lung outcomes of heifers fed 10-G

	TRT^*a*^			
Item	CON	10-G	SEM	*P-*value
Liver score,%				
Edible	81.53	84.54	—	0.17
Abscessed	12.26	9.23	—	0.10
A−	0.92	1.17	—	0.65
A	2.69	3.16	—	0.61
Total A+	8.51	5.27	—	0.04
A+	2.20	1.02	—	0.11
A + Open	3.38	2.15	—	0.20
A + Adhesion	1.32	0.80	—	0.29
A + Adhesion/Open	1.23	1.08	—	0.80
Total Other	5.99	5.85	—	0.91
Flukes	2.58	2.39	—	0.81
Telangiectasis	0.22	0.56	—	0.28
Contamination	2.06	2.08	—	0.98
Lung score^*b*^,%				
Normal	71.93	72.62	—	0.79
1	5.07	4.59	—	0.69
2	5.37	4.92	—	0.72
3	7.21	7.69	—	0.74
4	3.38	5.38	—	0.10
5	2.15	1.14	—	0.17
I	1.69	0.62	—	0.10
C	2.91	2.62	—	0.75

^*a*^Treatments included no DFM contained in the diet (CON) and a diet containing *L. acidophilus, E. faecium, P. pentosaceus, L. brevis*, and *L. plantarum* fed at 2 g/heifer/d providing 1 × 109 CFU (10-G) (Life Products, Inc., Norfolk, NE).

^*b*^N = normal; 1 = presence of mycoplasma-like lesion greater than 25%; 2 = plural adhesions, a portion of lung missing, or a combination of these affecting <25% of lung tissue; 3 = plural adhesions, a portion of lung missing, or a combination of these affecting >25% to 50% of lung tissue; 4 = plural adhesions, a portion of lung missing, or a combination of these affecting >50% to 75% of lung tissue; 5 = plural adhesions, a portion of lung missing, or a combination of these affecting >75% of lung tissue; C = contaminated; I = inflated.

The incidence of normal lungs (Table 3) was 72.62% and 71.93% for 10-G and CON cattle, respectively. Lungs scored 1, 2, 3, 5, or condemned did not differ (*P ≥ *0.17) between treatments. However, lungs with 50% to 75% adhesion or consolidation (score four) tended (*P = *0.10) to be more frequent in cattle fed 10-G (5.38%) when compared with CON (3.38%). Likewise, lungs that did not deflate at harvest tended (*P = *0.10) to occur more frequently in CON cattle (1.69%) over 10-G (0.62%).

### Carcass Performance

Hot carcass weight (Table 4) did not differ (*P = *0.14; CON = 370.5; 10-G = 374.2) between treatments. Several studies have reported no difference in HCW for cattle fed 10-G (Neuhold et al., 2012; Luebbe et al., 2013; Kenney et al., 2015). The dressed yield was similar (*P = *0.53) for 10-G and CON cattle (64.8% vs. 64.9%, respectively). Calculated empty body fat (*P =* 0.71), LMA (*P =* 0.13), marbling (*P = *0.20), and 12th-rib fat thickness (*P = *0.73) also did not differ between dietary treatments. Wilson et al. (2016) also reported no differences in 12th-rib fat thickness, LMA, and marbling score between control and DFM-fed cattle.

**Table 4.** Carcass performance of heifers supplemented with 10-G

	TRT^*a*^			
Item	CON	10-G	SEM	*P-*value
HCW, kg	370.5	374.2	4.9	0.14
Dressed yield, %	64.90	64.80	0.001	0.53
LMA, cm^2^	90.38	91.65	0.8	0.13
Marbling score^*b*^	506	516	12.4	0.20
12th-rib fat thickness, cm	1.88	1.89	0.05	0.73
Empty body fat^*c*^, %	32.00	32.11	0.35	0.71
Quality grade, %				
Prime	5.70	5.73	—	0.98
Choice	73.62	77.97	—	0.09
Select	18.94	14.64	—	0.06
Ungraded	0.62	0.27	—	0.71
Yield grade,%				
YG1	6.37	7.01	—	0.72
YG2	29.38	28.80	—	0.85
YG3	37.40	36.24	—	0.62
YG4	23.18	23.94	—	0.82
YG5	3.67	4.01	—	0.78

^*a*^Treatments included no DFM contained in the diet (CON) and a diet containing *L. acidophilus, E. faecium, P. pentosaceus, L. brevis*, and *L. plantarum* fed at 2 g/heifer/d providing 1 × 109 CFU (10-G) (Life Products, Inc., Norfolk, NE).

^*b*^300 = Slight, 400 = Small, 500 = Modest, and 600 = Moderate.

^*c*^Empty body fat calculated using EBF, % = 17.76107 + (4.68142 × FT) + (0.01945 × HCW) + (0.81855 × QG) − (0.06754 × LMA), where FT = 12th-rib fat thickness in cm, HCW = hot carcass weight in kg, QG = quality grade (4 = Select, 5 = Choice−, 6 = Choice, 7 = Choice+, and 8 = Prime), and LMA = longissimus muscle area in cm^2^ (Guiroy et al., 2001).

Percentage USDA Prime and Ungraded carcasses were not affected by supplementation of 10-G (*P ≥ *0.71). Heifers supplemented with 10-G tended (*P *= 0.06) to be represented by fewer USDA Select carcasses and more (*P = *0.09) USDA Choice carcasses when compared with CON cattle. Inconsistent effects of DFM supplementation on USDA quality grades have been shown with varied results. Huck et al. (2000) and Thompson et al. (2020) reported a similar tendency for an increased percentage of USDA Choice carcasses when fed a DFM compared with non-supplemented cattle, while other studies in which cattle were supplemented with a DFM have shown no improvement in USDA quality grade distribution (Elam et al., 2003; Krehbiel et al., 2003; Cull et al., 2015). Yield grade distributions did not differ between treatment groups (*P *≥ 0.62). Dick et al. (2013) fed various concentrations (0, 1 × 10^5^, 1 × 10^6^) of *L. acidophilus* and *Propionibacterium freudenreichii* to calf-fed Holstein steers and reported similar yield grade for all three concentrations.

### *Salmonella* prevalence

#### Fecal pats.

*Salmonella* prevalence in fecal pats (Table 5) did not differ (*P = *0.73) between dietary treatments at day 0, 52, or 191. However, at day 120, fecal pats from heifers fed 10-G tended (*P* = 0.09) to have 20% lower *Salmonella* prevalence. Brown et al. (2020) reported that non-supplemented cattle and those supplemented with either a 1:1 ratio of *L. acidophilus* and *P. acidilactici* or a 1:2 ratio of *Lactobacillus reuteri* and other *Lactobacillus* strains were 90.0%, 86.7%, and 100.0% positive for *Salmonella.*

**Table 5.** *Salmonella* prevalence and log of composited fecal pats collected from cattle fed 10-G

	TRT^*a*^		SEM	*P-*value		
Item	CON	10-G		TRT	TIME	TRT × TIME
*Salmonella* prevalence, % positive			—	—	<0.01	0.36
Day 0	14.0	18.0	—	0.73		
Day 52	96.0	100.0	—	0.73		
Day 120	88.0	68.0	—	0.09		
Day 192	78.0	82.0	—	0.73		
*Salmonella* log, MPN/g	—	—	0.28	—	<0.01	0.49
Day 0	0.40	0.86	0.56	0.41	—	—
Day 52	4.22	4.84	0.56	0.28	—	—
Day 120	3.08	2.68	0.56	0.48	—	—
Day 192	3.23	2.99	0.56	0.66	—	—

^*a*^Treatments included no DFM contained in the diet (CON) and a diet containing *L. acidophilus, E. faecium, P. pentosaceus, L. brevis*, and *L. plantarum* fed at 2 g/heifer/d providing 1 × 10^9^ CFU (10-G) (Life Products, Inc., Norfolk, NE).

A time effect (*P < *0.01) was observed across the sampling periods in the current study. *Salmonella* was present in 16% of all samples at 0 d in March and increased to 98% at 52 d in May, whereas prevalence from samples taken at 120 d in July and 191 d in September was similar (78.0% and 80.0%, respectively). *Salmonella* concentration did not differ (*P *≥ 0.28) between treatments but followed the same pattern as prevalence rates with an initial log of 0.63 mpn/g followed by a significant increase before plateauing at a log of 3.11 mpn/g at 192 d. The significant increase in log concentration is most likely due to environmental seasonality. *Salmonella* is known to increase in warmer months and decline in cooler months. Barkocy-Gallagher et al. (2003) reported *Salmonella* prevalence to be more than 3.5-fold greater in summer compared with prevalence in fall, winter, and spring. Dargatz et al. (2003) also reported that warm-season months were significantly higher for *Salmonella* prevalence when compared with cool season.

#### Rectoanal mucosal swabs.

*Salmonella* prevalence of RAMS (Table 6) was similar at day 0 (*P* = 0.97; 11.6%) and also did not differ (*P *= 0.91) between 10-G (99.0%) and CON cattle (98.6%) at the end of the finishing period. Tabe et al. (2008) reported a numerically lower percentage for overall *Salmonella* prevalence in fecal grabs (12.7%) from both non-supplemented or DFM-supplemented cattle. Conversely, *Salmonella* shedding increased throughout the feeding period leading to an increasing trend in *Salmonella* prevalence from the initial sampling.

**Table 6.** *Salmonella* prevalence and log of rectoanal mucosal swabs of heifers supplemented with 10-G

	TRT^*a*^			*P-*value		
Item	CON	10-G	SEM	TRT	TIME	TRT × DAY
*Salmonella* prevalence,%			—	—	<0.01	0.95
Day 0	11.5	11.6	—	0.97	—	—
Day 192	99.0	98.6	—	0.91	—	—
*Salmonella* log (MPN/g)			0.39	—	<0.01	0.59
Day 0	0.28	0.30	0.48	0.97	—	—
Day 192	4.40	4.05	0.48	0.47	—	—

^*a*^Treatments included no DFM contained in the diet (CON) and a diet containing *L. acidophilus, E. faecium, P. pentosaceus, L. brevis*, and *L. plantarum* fed at 2 g/heifer/d providing 1 × 10^9^ CFU (10-G) (Life Products, Inc., Norfolk, NE).

*Salmonella* prevalence increased (*P < *0.01) dramatically from initial processing in March (11.55%) to harvest (98.80%) in September. The increase in prevalence observed can be largely attributed to the seasonal nature of when samples were collected. Other researchers have described a seasonal effect with prevalence rates peaking in warmer months and troughing in cooler months (Barkocy-Gallagher et al., 2003; Gragg et al., 2013a; Webb et al., 2017). The increase in the prevalence of *Salmonella* would suggest that environment is an important component for *Salmonella* harborage in peripheral lymph nodes, especially in regions where it is consistently warm.

Overall log concentration (mpn/g) between dietary treatments did not differ at day 0 (*P = *0.97; 10-G = 0.30, CON = 0.28) or at harvest (*P* = 0.47; CON = 4.4, 10-G = 4.1) but increased (*P* < 0.01) more than 14-fold during the 192-d study.

#### Lymph nodes.

Salmonella prevalence and concentration data are presented in Table 7. Cattle fed 10-G had a lower frequency (*P *= 0.01; 7.42%) of *Salmonella* positive lymph nodes when compared with CON (15.80%). Vipham et al. (2015) supplemented cattle with *L. acidophilus* and *P. freudenreichii*, which resulted in an 18.8% reduction of *Salmonella* in subiliac lymph nodes. In addition, Brown et al. (2020) fed different blends of bacterial DFMs and reported that control cattle tended to have a numerically greater percent-positive of *Salmonella* in peripheral lymph nodes when compared with treated cattle, suggestive of a potential treatment effect. Concomitantly, *Salmonella* concentration (mpn/g) of the lymph nodes did not differ (*P* = 0.34) between dietary treatments (10-G = 0.34, CON = 0.73) of all samples. Additionally, DFMs may elicit stimulation or alteration of the immune system as DFM treatments had improved immune function via suppressed or downregulated innate immunity and differences in pathogen prevalence. Webb et al. (2017) reported log concentrations ranging from 1.6 to 4.9 log_10_ CFU/PLN from 160 quantifiable subiliac lymph nodes collected.

**Table 7.** *Salmonella* prevalence and log of subiliac lymph nodes collected from heifers fed 10-G

	TRT^*a*^			*P-*value
Item	CON	10-G	SEM	TRT
*Salmonella*, % positive	15.80	7.42	—	0.01
*Salmonella* log, MPN/g (mean of all samples)	0.73	0.34	0.28	0.34
*Salmonella* log, MPN/g (mean of positive samples)	3.30	3.75	0.94	0.65
*Salmonella*, minimum log MPN/g	0.00	0.00	—	—
*Salmonella*, maximum log MPN/g	6.54	5.64	—	—

^*a*^Treatments included no DFM contained in the diet (CON) and a diet containing *L. acidophilus, E. faecium, P. pentosaceus, L. brevis*, and *L. plantarum* fed at 2 g/heifer/d providing 1 × 10^9^ CFU (10-G) (Life Products, Inc., Norfolk, NE).

## CONCLUSION

These data indicate that supplementation of 10-G had no influence on feedlot performance or carcass traits. Conversely, feeding 10-G may directly benefit the producer via improved carcass grading outcomes and reduced frequency of severe liver abscesses. Supplementation of 10-G did significantly reduce the frequency of *Salmonella* positive lymph nodes, which may translate into improved public health outcomes by reducing the number of foodborne illnesses caused by *Salmonella*.
